# Delayed recognition of an ipsilateral femoral neck and shaft fracture leading to preventable subsequent complications: a case report

**DOI:** 10.1186/s13037-017-0134-0

**Published:** 2017-07-10

**Authors:** Sönke Labza, Isabella Fassola, Benedict Kunz, Wolfgang Ertel, Senat Krasnici

**Affiliations:** 1 Klinik für Unfallchirurgie und Orthopädie, DRK Kliniken Berlin | Westend, Berlin, 14050 Germany; 2 Klinik für orthopädische und Unfallchirurgie, CHARITÉ - Campus Benjamin Franklin, Hindenburgdamm 30, 12200 Berlin, Germany

**Keywords:** Polytrauma, Missed injury, Ipsilateral, Femur, Femoral neck, Femoral shaft, Fracture

## Abstract

**Background:**

Ipsilateral femoral shaft and neck fractures are rare injuries, affecting mostly young patients who sustained high-energy traumas. In 19–50% of cases, the femoral fracture is misdiagnosed or overlooked at the initial presentation, with reportedly increased risk of complications such as non-union and avascular necrosis. We present a case of an ipsilateral femoral neck and shaft fracture, which was missed at initial presentation despite radiographic and computed tomography (CT) scan evaluation.

**Case presentation:**

A 56-year old female was admitted to our institution following a high-energy trauma (fall from 6 m). Initial radiographic and CT scan evaluation revealed a displaced femoral shaft fracture but no other femoral fractures were detected. Closed reduction and external fixation of the femoral shaft fracture was performed in the emergency setting. Follow-up radiologic evaluations revealed an ipsilateral laterally displaced femoral neck fracture. Despite cephalomedullary nail fixation of both fractures performed on the third day from the initial injury, the patient developed a non-union of the femoral neck fracture, which led to cut-out of the lag screw with associated varus failure of the femoral neck fracture requiring surgical revision and implant of a bipolar hemiarthroplasty at one year follow up. The postoperative course was uneventful and the patient had a full long-term recovery.

**Conclusion:**

This case report exemplifies the need to maintain the highest level of suspiciousness for the concomitant presence of an ipsilateral femoral neck fracture when treating polytraumatized patients who sustained a femoral shaft fracture as a consequence of a high-energy trauma. Furthermore, the pre-operative standardized radiological evaluation (plain x-ray and CT scan) might not always help in ruling out these fractures. It is therefore necessary to adopt additional standardized radiographic protocols not only in the pre-operative but also in the intra-operative and immediate post-operative settings.

## Background

Ipsilateral femoral shaft and neck fractures are uncommon injuries [1–5]. The incidence is approximately 0.8 to 9% of all femoral shaft fractures [2, 3, 6–15]. Most of the patients are young adults, who sustained multiple injuries caused by high-energy trauma such as traffic accidents and jumping or falling from heights [2, 4, 7–13, 16–18]. Ipsilateral shaft and neck fractures in the elderly may occur after low-energy injuries [3, 9, 13, 19–22]. We report our experience with a patient affected by femoral shaft and neck fractures and we discuss the proposed diagnostic algorithms, the treatment approaches, and the complications presented in the literature.

## Case presentation

**Fig. 1:**
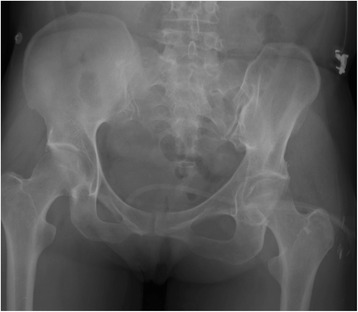
Pre-operative anteroposterior radiograph of pelvis demonstrating lack of evidence of left femoral neck fracture

**Fig. 2:**
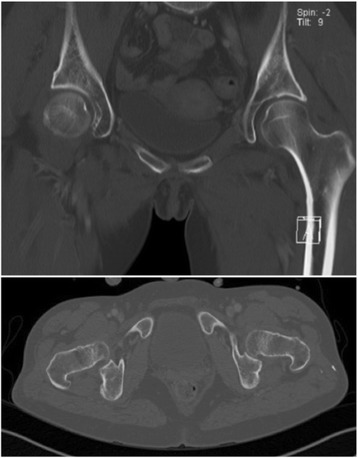
Pre-operative CT coronal and axial views of pelvis and of the proximal femur did not show clear evidence of fracture

**Fig. 3:**
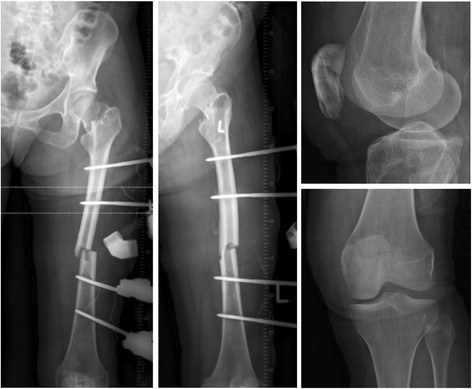
Follow-up radiograph of pelvis at day 1 post-op, demonstrating displacement of the missed left femoral neck fracture and an ipsilateral patella fracture

**Fig. 4:**
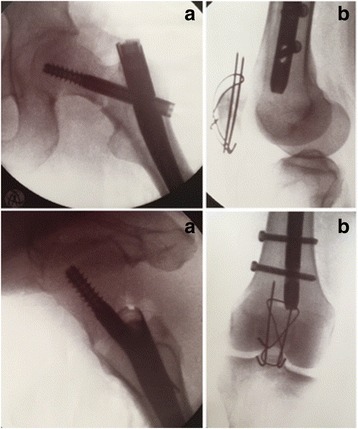
Intra-operative fluoroscopic control of **a**) the cephalomedullary nail, demonstrating a tip-apex-distance of 10 mm and a central position of the lag screw; **b**) the reduction and osteosynthesis of the patella fracture with a standard figure-of-8 tension band wire

**Fig. 5:**
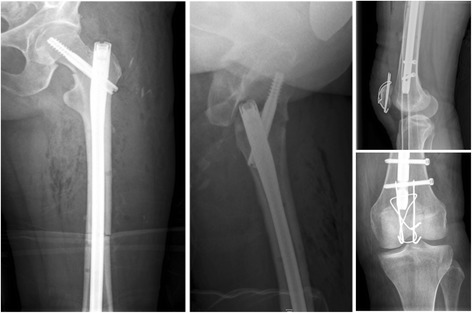
Follow-up radiographs of left hip at day 3 post-op, showing satisfactory reduction and fixation of the left femoral neck and shaft fractures by intramedullary nail and the patella fracture, respectively

**Fig. 6:**
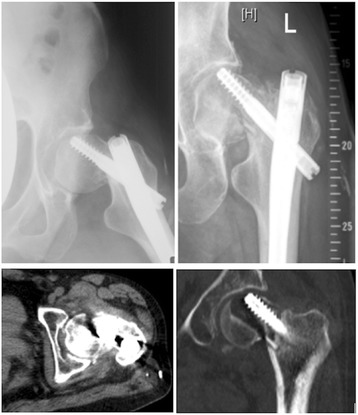
Follow-up radiographs (5 months post-op) and CT scan (7 months post-op) of pelvis and left hip, demonstrating lag-screw cut-out with displacement of the femoral neck fracture

**Fig. 7:**
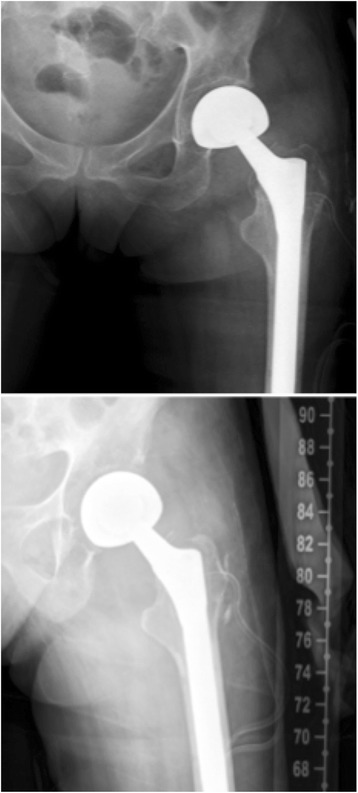
Post-operative (12 months) AP pelvic x-ray showing the bipolar hemiarthroplasty following removal of the cephalomedullary nail

The patient was a 56-year old female, who sustained several injuries during a fall from 6 m (third floor). Glasgow Coma Scale (GCS) on admission was 6–7/15. Injury Severity Scale (ISS) score was 34 [Table 1]. She was intubated, stabilized and admitted. The initial diagnostic evaluation was performed according to Advanced Trauma Life Support (ATLS) guidelines and included Focused Assessment with Sonography in Trauma (FAST), chest x-ray and pelvic antero-posterior (AP) x-rays, CT-trauma scan and femoral x-rays. Trauma workup revealed diffuse intracerebral contusion, a small parieto-occipital subarachnoid hemorrhage on the right, bilateral mediastinum and lung contusion, maxillary-orbital and temporal bone fractures, the radiological examination revealed a femoral shaft fracture on the left. No other femoral fractures were detected upon X-ray examination [Fig. 1]. CT coronal and axial views of the pelvis (3 mm thickness) and of the proximal femur did not show clear evidence of fracture [Fig. 2]. Initial treatment, on the day of admission, consisted of closed reduction and external fixation of the femoral shaft. During surgical treatment of the maxillary fractures on post-injury day 1, it was noted that the left lower extremity was shortened and externally rotated. Plain films of the left hip, femur, and knee demonstrated a displaced femoral neck facture and an ipsilateral patellar fracture [Fig. 3]. On post-injury day 3, the left femoral shaft and neck fracture was treated with a cephalomedullary nail on a standard traction table by closed reduction. Anatomic reduction and stable fixation of the femur fractures were ascertained intra-operatively by fluoroscopic control, revealing a tip-apex-distance of 10 mm and a central position of the lag screw. The patella fracture was treated with a standard figure-of-8 tension band wire [Fig. 4]. Post-operative radiographic control confirmed the satisfactory reduction and stabilization of the left femoral neck and shaft fracture as well as of the ipsilateral patella fracture [Fig. 5]. The post-operative course was uneventful. The patient was transferred to a rehabilitation center four weeks after admission and had a physiotherapy exercise protocol with weight bearing on the left lower extremity up to max 10–15 kg (kg) for 6 weeks post-operatively. Full weight-bearing was allowed after 12 weeks. Sport activities were restricted for 6 months. At post-operative weeks 9 and 16, the patient complained of pain in the left groin and shaft, and radiographs demonstrated callous formation in the left femoral shaft. X-rays at post-operative week 28 demonstrated lag-screw cut-out with displacement of the femoral neck fracture [Fig. 6]. The patient was offered surgical revision but she refused at this time. One year after injury, the patient agreed to revision surgery and on week 52 (1 year) post-injury she underwent removal of the cephalomedullary implant and placement of a bipolar hemiarthroplasty after being offered a total hip replacement, which she refused. Post-operative clinical and radiological controls were unremarkable [Fig. 7]. Post-operative course was uneventful and the patient was transferred to a rehabilitation center two weeks after the surgery.

**Table 1:** List of patient’s injuries and ISS

Region	Injury description	AIS score	top three^2^
Head or neck	diffuse intracerebral contusion	3	
	**traumatic right parieto-occipital** **subrachnoid hemorrhage**	**4**	**16**
Face	left zygomatic bone fracture	3	
	right temporal fracture	3	
	right orbital rim fracture	3	
	right orbital floor fracture	3	
Chest	mediastinal contusion	2	
	**bilateral lung contusion**	**3**	**9**
Abdomen or pelvis contents	no injury	0	
Extremities or pelvic girdle	**left femoral shaft fracture**	**3**	**9**
	left patella fracture	2	
External	alcohol intoxication	2	
	traumatic rhabdomyolysis	3	
	acute hemorrhagic anemia	3	
	hypokalemia	3	
**ISS (Σ 3most AIS score)** ^**2**^			**34**

Only the highest AIS score in each body region is used (highlighted in Bold). The 3 most severely injured body regions have their score squared and added together to produce the ISS score

## Discussion

Overlooked or delayed diagnoses are commonly reported in literature when treating polytraumatized patients (1.3 to 39%) [23, 24]. In case of ipsilateral femoral neck and shaft fractures, the delayed diagnosis of the femoral neck fracture occurs in 19 to 50% of patients during the initial examination [2, 7, 8, 11, 12, 19, 25–28]. According to the literature, several factors may account for the pre-operative mis- or delayed diagnosis of ipsilateral femoral shaft and neck fractures. Among others, the main factor includes the type of fracture (nondisplaced or minimally displaced in 26 to 59% of cases) [2, 29], as in our case. The pre-operative hip and pelvic radiographic exams were initially deemed negative for a femoral neck fracture since no displacement was evident. As the patient was already intubated upon arrival, collection of symptoms supporting suspicion of a femoral neck fracture was minimal during early clinical examination. The treatment was focused on life-threatening and obvious injuries. The particularities of the treatment of a multiply injured patient, in addition, may play a role in the delayed diagnosis [11, 12, 27]. Several studies show that an insufficient radiological protocol seems to affect the incidence of mis- or delayed diagnosis [2, 4, 11–14, 18, 26, 28, 30–33].

In our case the patient was evaluated with a pre-operative AP pelvis, which was negative. It is unclear whether a lateral view of the hip could have been more sensitive in detecting the femoral neck fracture. Nonetheless, we tend to agree with the statement that a dedicated antero-posterior internal rotation hip radiograph, performed intra-operatively or immediately after the reduction and stabilization of the femoral shaft fracture, could have improved the likelihood of detecting the fracture of the femoral neck, by minimally displacing the femoral neck fracture and making the diagnosis less difficult [28].

The diagnostic value of the preoperative CT scan is still controversial. Some authors claim that its use helps reducing the delay in diagnosis of femoral neck fracture (from 57 to 6.3%) [26, 28, 29, 34, 35]. Others claim that the significance of CT is equivalent of that of the plain radiography (sensitivity of only 56% to 64) [29, 36, 37]. In our case the emergency CT scan available offered coronal and axial views with thickness of 3 mm. Perhaps using thin-cut computer tomography CT scan (thickness 1–2 mm) could have improved the ability to detect the non-displaced femoral neck fractures. Combining different preoperative (thin-cut computed tomography CT scan and dedicated antero-posterior internal rotation radiographs of the femoral neck, including 2D CT reconstructions), intraoperative (lateral hip fluoroscopic view by angulation of the radiographic beam before reducing the shaft fracture or plain radiograph view centered at the hip with 10° to 15° of hip internal rotation following fixation) and postoperative (dedicated AP internal rotation views of the hip) clinical and radiological measures should help reduce the incidence of a missed femoral neck fracture.

In our case a pre-operative CT was performed according to the protocol and no evidence of femoral neck fracture was found. We agree with other authors that CT scan even when associated with plain radiographs should not be considered as an unreserved assessment tool [36, 37] and that intra-operative maneuvers and radiographs should be used to rule out concomitant femoral neck fractures.

Variable rates of complications and results have been reported in patients suffering from ipsilateral femoral neck and shaft fracture [3, 9, 13, 16, 38]. Common complications (incidence 4 to 22%) of the femoral head are aseptic necrosis [5, 11, 12, 31] and nonunion [11, 12, 31, 39–41]. Complications of the shaft fractures are nonunion caused by an open fracture, inadequate implant (nail diameter too small), no reaming and prolonged delay to weight bearing [2, 3]. Clear evidence that a delayed diagnosis of femoral neck fracture in these complex injuries affects the incidence of complications such as non-union and avascular necrosis [2, 3, 7, 8, 19] is still lacking. According to some authors, the delayed diagnosis of femoral neck fracture in these complex injuries does not seem to affect the incidence of complications such as non-union and avascular necrosis [2, 19]. Conversely others report that the risk of healing complications is higher in late surgery compared to early surgery and in combined shaft and neck fractures compared to one-level-injuries [3]. In the present case report the delay between diagnosis and treatment of the ipsilateral femoral neck and shaft fracture was 2 days, making it impossible to state with certainty whether or not the delay in diagnosis is responsible for the non-union. Timely recognition and early surgical treatment of ipsilateral femoral shaft and neck fractures are crucial for early mobilization and rehabilitation, allowing overall good functional outcomes [6, 17] and reduces mortality and morbidity [25]. Deciding on the appropriate therapy remains challenging [3]. Different strategies for the treatment of ipsilateral femoral neck and shaft fractures have been proposed: cannulated screws for the femoral neck and a retrograde locking nail for the femoral shaft [4, 35, 42], cephalomedullary implant [7–9, 20, 43], antegrade nail with one neck screw [6, 13, 20, 22, 44, 45], long proximal femoral nail [46, 47]. At the present time, however, there is still no consensus on the superiority of a treatment protocol [17, 48, 49]. In the present case, we opted to treat both fractures with antegrade nail with one neck screw and the timing for the surgery was within one week from the traumatic event. The intra- and post-operative radiographs excluded the presence of a reduction in varus and implant placement was deemed satisfactory, although a smaller tip-apex distance could have provided a better purchase in subchondral bone and therefore possibly reduce the risk of early cut-out [28].

## Conclusion

In polytraumatized patients with injuries caused by high-energy trauma one must expect and rule out combined injuries of the femur at different levels. Despite the uncommon presentation of this complex fracture pattern, the knowledge and awareness of this complex injury pattern should be increased in every Trauma Center. The use of specific standardized protocols for the correct diagnosis and treatment of such fractures in order to decrease the incidence of devastating complications should be implemented. The present case clearly shows that even the use of a preoperative CT scan in addition to routine pelvic and hip joint radiograph might sometimes lead to a misdiagnosis. It is therefore important to understand that, especially in polytraumatized, non-collaborating patient who present with femoral shaft fracture, the highest level of suspiciousness must be maintained for the concomitant presence of an ipsilateral femoral neck fracture. Thus the combination of specific radiographic preoperative, intraoperative and postoperative views of the femoral neck should be integrated in the ATLS algorithm of the polytraumatized patient [26] to help reduce the incidence of a missed femoral neck fracture.

## Abbreviations

2D CT: Two dimensional computerized tomography
AIS: Abbreviated injury score
AP: Antero-posterior
ATLS: Advanced trauma life support
CT: Computed tomography
FAST: Focused assessment for sonography in trauma
GCS: Glasgow coma scale
ISS: Injury severity score
